# Glycosylation: Rising Potential for Prostate Cancer Evaluation

**DOI:** 10.3390/cancers13153726

**Published:** 2021-07-24

**Authors:** Anna Kałuża, Justyna Szczykutowicz, Mirosława Ferens-Sieczkowska

**Affiliations:** Department of Chemistry and Immunochemistry, Wroclaw Medical University, Sklodowskiej-Curie 48/50, 50-369 Wroclaw, Poland; justyna.szczykutowicz@umed.wroc.pl (J.S.); miroslawa.ferens-sieczkowska@umed.wroc.pl (M.F.-S.)

**Keywords:** glycosylation, glycans, prostate cancer, prostate specific antigen, biomarkers

## Abstract

**Simple Summary:**

Aberrant protein glycosylation is a well-known hallmark of cancer and is associated with differential expression of enzymes such as glycosyltransferases and glycosidases. The altered expression of the enzymes triggers cancer cells to produce glycoproteins with specific cancer-related aberrations in glycan structures. Increasing number of data indicate that glycosylation patterns of PSA and other prostate-originated proteins exert a potential to distinguish between benign prostate disease and cancer as well as among different stages of prostate cancer development and aggressiveness. This review summarizes the alterations in glycan sialylation, fucosylation, truncated O-glycans, and LacdiNAc groups outlining their potential applications in non-invasive diagnostic procedures of prostate diseases. Further research is desired to develop more general algorithms exploiting glycobiology data for the improvement of prostate diseases evaluation.

**Abstract:**

Prostate cancer is the second most commonly diagnosed cancer among men. Alterations in protein glycosylation are confirmed to be a reliable hallmark of cancer. Prostate-specific antigen is the biomarker that is used most frequently for prostate cancer detection, although its lack of sensitivity and specificity results in many unnecessary biopsies. A wide range of glycosylation alterations in prostate cancer cells, including increased sialylation and fucosylation, can modify protein function and play a crucial role in many important biological processes in cancer, including cell signalling, adhesion, migration, and cellular metabolism. In this review, we summarize studies evaluating the prostate cancer associated glycosylation related alterations in sialylation, mainly α2,3-sialylation, core fucosylation, branched N-glycans, LacdiNAc group and presence of truncated O-glycans (sTn, sT antigen). Finally, we discuss the great potential to make use of glycans as diagnostic and prognostic biomarkers for prostate cancer.

## 1. Introduction

Prostate cancer was the second most commonly occurring cancer and the fifth leading cause of cancer death among men in 2020 [1,2]. In 1986, prostate-specific antigen (PSA) was approved by the United States Food and Drug Administration (FDA) to screen men for prostate cancer (PCa). PSA is present in small quantities in the serum of men with a healthy prostate (up to 2.5 ng/mL), whereas a concentration above 4 ng/mL is considered indicative of prostate cancer or benign prostatic hyperplasia (BPH) [3,4]. Frequently, PSA screening for prostate cancer has limited sensitivity and specificity, which can lead to overdiagnosis and overtreatment of indolent disease, resulting in unnecessary, invasive biopsy and treatments for non-aggressive cancers [5,6]. Hence, in the following years serum PSA screening in association with digital rectal exam (DRE) and Gleason scoring of prostate biopsy samples was approved by the FDA for the early detection of prostate cancer [7]. Subsequent approaches for refining the specificity and sensitivity of the serum PSA test involved calculating the proportion of free PSA (fPSA) to total PSA (tPSA), and PSA complexed with alpha-1-antichymotrypsin and α2-macroglobulin to total PSA [7], as well as focusing on the individual molecular forms of PSA such as proPSA, benign PSA, and intact PSA. A multifactorial PSA test, such as the Prostate Health Index (PHI) and the four-kallikrein panel (4Kscore), can improve diagnostic accuracy but has limited prognostic usefulness, and may miss some aggressive tumors [8,9,10]. More recently, imaging techniques such as multiparametric magnetic resonance imaging (mpMRI) and prostate-specific membrane antigen (PSMA) positron emission tomography (PET)/computed tomography (CT) have evaluated as a potential tool for staging prostate cancer in men before radical treatment. However, the limitations of both techniques were identified. Detection of clinically significant PCa in multiparametric MRI is changeable and depends on a few factors, including tumor location and volume, Gleason score, moreover the analysis of multiparametric prostate MRI is operator dependent [11]. Meanwhile, the sensitivity of PSMA PET/CT technique for the detection of metastases is reduced, presumably due to limitations in the spatial resolution of detecting small tumor deposits in primary and recurrent prostate cancer [12]. Researchers also compared the clinical utility of prostate cancer antigen 3 (PCA3) with PSA in a serum test [13] and widely examined PSA post-translational modification such as glycosylation [14,15,16]. Due to the high importance of glycan alterations observed in prostate cancer, including increased sialylation and core fucosylation, the emergence of truncated O-glycans and branched N-glycans, in this review we discuss the great potential to make use of glycans as diagnostic and prognostic biomarkers for prostate cancer. Our review focuses on PSA, the glycoprotein that has been studied in the most detail so far, but the variety of scientific approaches is remarkable, therefore unfractionated biological material such as whole serum were also considered. Not all glycoepitopes important for prostate cancer proliferation have been observed for PSA, thus analyzing only a selected glycoprotein, a lot of data relevant to the assessment of glycosylation changes in prostate disease may be lost. Therefore, presented work does not provide evident solutions for the selection of glycoepitopes that could effectively act as prognostic biomarkers, the studies available so far do not allow for such far-reaching conclusions. 

## 2. Action of Androgens in the Prostate Gland

The biomolecules secreted in prostatic fluid are engaged in the regulation of prostate epithelium homeostasis and the process of ejaculation. The agents that provide these functions are kallikreins, which include PSA and kallikrein-related peptidase 2 (hK2). The others are citrate, an intermediate product of the Krebs cycle, and Zn^2+^, a chemical element actively stored within the cytoplasm of the prostatic epithelial cells [17,18]. Physiological growth and development of prostatic epithelium, as well as the regulation of its secretory functions, depend on the appropriate action of androgen steroid hormones [19]. The action of androgens is mediated via the androgen receptor (AR), a ligand-activated transcription factor and member of the steroid hormone nuclear receptor family, which mediates androgen signaling by binding to androgen response elements (AREs) in both normal prostate tissue and prostate cancer [20,21,22].

Healthy prostate cells store the largest amount of zinc ions of all soft tissues in the human body. This remarkable property is based on the fact that prostatic epithelial cells retain Zn^2+^ through androgen-dependent Zn^2+^ cellular uptake and maintain the cycle in which specific zinc transporters are involved [17,18]. The accumulation of Zn^2+^ and citrate within the prostate, suppression of the Krebs cycle, and the release of fluid from the gland are regulated by androgens. In prostate tissue, a more potent derivative 5α-dihydrotestosterone (DHT), synthesized from testosterone by 5α-reductase enzyme, is the primary ligand for the AR [23]. Hundreds of genes have been identified that are regulated via DHT in prostate epithelial cells, many of which are essential genes involved in the maintenance of prostate homeostasis [24,25]. Circulating testosterone level and intraprostatic DHT concentration decrease gradually with ageing, causing the gland to malfunction by reducing its ability to sustain a healthy tissue level of intracellular Zn^2+^, KLK-secreted proteins and citrate within the prostate liquid.

Normal prostate epithelial cells display characteristic behaviour in relation to their metabolic pathway. They are programmed to produce citrate, unlike most cells which oxidize it [26]. The inhibition of the Krebs cycle in normal prostate cells results in the accumulation and subsequent secretion of citrate as a component of semen. This distinctive process of citrate production is supported by another specific feature of the prostate epithelial cells: the ability to store high concentrations of zinc, which have been shown to inhibit m-aconitase, the enzyme that catalyses the oxidation of citrate in the Krebs cycle [27,28]. Accumulation of zinc in prostate epithelium is driven by an increased amount of the zinc transporter ZIP1 in tissue [27,28]. In normal prostate epithelial cells, through the accumulation of zinc, the Krebs cycle is inhibited, so these cells are energetically inefficient. This leads to an ATP production process different than present in most cells [29]. In contrast, prostate cancer cells reverse this process, leading to reactions that cause zinc loss and citrate oxidation, which is a major change in energy metabolism [18,28]. It was discovered long ego that prostate cancer cells do not exhibit the standard Warburg effect, observed in most cancer cells, whereby high glucose uptake and lactate release are considered hallmarks of most tumors [30]. As opposed to most cancer cells that utilize aerobic glycolysis, prostate cancer cells show a higher level of citric acid cycle activity compared to normal cells [26,28,31].

Apart from that, androgens are crucial for the development and metastatic progression of prostate cancer. Undoubtedly, androgen deprivation therapy (ADT) is the widely accepted initial treatment for symptomatic metastatic prostate cancer. In the early stages of diseases ADT is effective, but after 2–3 years the patients may develop castration-resistant prostate cancer (CRPC), which is ultimately lethal [32,33].

## 3. Prostatic Inflammation, Benign Prostatic Hyperplasia and Prostate Cancer 

Recent data support the role of chronic prostatic inflammation as a predisposing factor for development of BPH [34] and prostate cancer [35]. Several authors have highlighted the significance of prostatic inflammation in the origin of BPH, pointing out that the prostate is an immunocompetent gland in which a small number of inflammatory cells such as T and B lymphocytes, mast cells and macrophages are physiologically present [36]. In adults, chronic inflammatory prostate infiltrates vary greatly in BPH and healthy tissues. The most common cells of infiltrates from patients with BPH are CD19^+^ or CD20^+^ B lymphocytes, CD4^+^ T lymphocytes and macrophages [37]. The B and T cells as well as macrophages occurring in the adult prostate can contribute to the damage of both epithelial and stromal cells, induce cytokine production and increase the concentration of growth factors that can stimulate an anomalous remodelling process. Interestingly, IL-8 has been suggested as a link between chronic prostate inflammation and the development of BPH. Several studies have indicated significantly elevated IL-8 expression in epithelial prostate cells and emphasized that this can trigger the expression of fibroblast growth factor (FGF) and stromal-epithelial growth factor signaling and subsequently induce abnormal proliferation of prostatic cells [38]. In consequence, tissue damage can initiate a chronic wound healing process that can induce prostate enlargement, potentially resulting in BPH [36].

## 4. Biology of Prostate-Specific Antigen

Prostate fluid is a rich source of prostate-derived proteins that can be used for biomarker discovery in a number of prevalent benign and malignant prostatic diseases including prostatitis, benign prostatic hyperplasia and prostate cancer. Several glycosylated proteins have been accepted as cancer biomarkers by the FDA, including prostate-specific antigen [39,40]. Prostate-specific antigen (PSA), also known as human kallikrein 3 (KLK3), is currently widely used as the gold standard biomarker for screening and diagnosis of prostate cancer [4]. PSA is an organ-specific glycoprotein, secreted by the epithelium and periurethral glands. PSA occurs as a 237-amino acid serine protease with chymotrypsin-like activity, and its transcription is regulated by androgens [41,42]. PSA is secreted as an inactive proenzyme (proPSA) into seminal fluid, which during the liquefaction process is converted by kallikrein-related peptidase 2 to a 33 kDa mature active form [3,43,44]. In the early stage of prostate cancer development, disruption of the basal cell layer and the basement membrane of the prostate epithelium results in leakage of PSA into the peripheral circulation, and this circulating blood form of PSA is identified in the early detection tests for prostate cancer [3]. The serum PSA level increases with the clinical severity of disease and is proportional to tumor volume, whereas after radical prostatectomy its level in serum reaches undetectable values [41,45]. Schroeder et al. showed that PSA-based screening of prostate cancer reduced the rate of death by 20% but simultaneously was correlated with a high degree of overdiagnosis [46].

PSA has a single N-glycosylation site at asparagine (Asn-69), verified in X-ray crystal structures [47,48]. In 2013, the results of a comprehensive multi-laboratory ABRF study examining N-glycosylation of Asn-69 in healthy donors were announced, and they made it possible to describe the structure of glycans attached to PSA [3,48,49]. The most abundant glycans identified were four biantennary glycan structures, consisting of three mannose, two galactose subunits, and four β-N-acetylglucosamine residues, estimated to comprise approximately 80% of the total number of PSA-related glycans. These all have one or two terminal sialic acid residues and the presence or absence of a core fucose. Two dominant hybrid structures have also been reported [3,48,49].

## 5. Glycosylation Changes in Cancer

**Figure 1:**
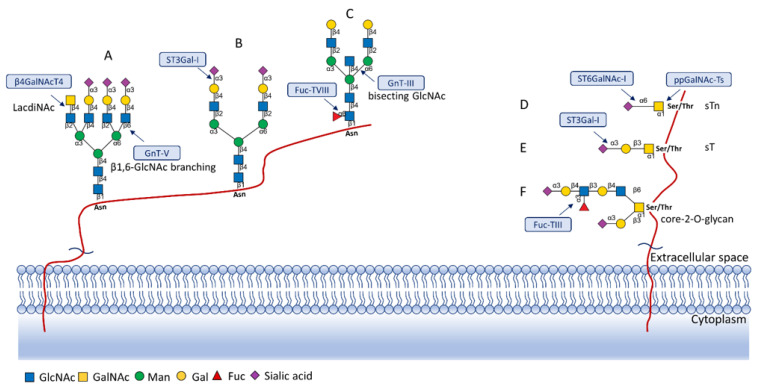
Overexpressed glycan structures typical for PCa. (**A**) Highly branched (tetra-antennary) N-glycan with LacdiNAc motif; (**B**) Biantennary N-glycan terminated with α2,3-linked SA; (**C**) Biantennary N-glycan with core fucose and bisecting GlcNAc; (**D**) sTn truncated O-glycan; (**E**) sT O-glycan antigen; (**F**) core-2-O-glycan with sLe^X^ antigen. Blue boxes indicate glycosyltransferases involved in the synthesis of particular structures.

Protein glycosylation is a post-translational process that enhances molecular heterogeneity as well as functional diversity within cell populations. Tumor cells show a wide range of glycosylation rearrangement in relation to their unchanged counterparts. Hakomori and Kannagi were the first to postulate the two main mechanisms underlying the cancer-associated changes of carbohydrate structures, called incomplete synthesis and neo-synthesis [50]. The incomplete biosynthesis pathway, typical for the early stages of a tumor, is the result of defective synthesis of typical glycans present in normal epithelial cells, leading to the synthesis of truncated structures such as short-chain O-GalNAc glycans (Tn, T, sialyl-Tn, and sialyl-T antigens). In contrast, neo-synthesis occurs extensively in advanced stages of cancer, and is associated with the induction of some genes involved in the glycosylation pathway, which results in de novo expression of antigens such as Lewis blood group related antigens (Le) and their sialylated counterparts: sialyl Lewis A (sLe^a^) and sialyl Lewis X (sLe^x^) antigens (Figure 1) [50,51]. Altered expression of glycans may result from several biological factors: (1) overexpression or underexpression of the relevant glycosyltransferases in the Golgi apparatus, (2) changed glycosidase activity, (3) alterations in tertiary peptide backbone composition, and (4) the availability and sufficiency of the sugar nucleotide donors and cofactors [52,53,54]. The presence and molecular density of the glycans affect the half-life of many different types of membrane receptor proteins, including glucose transporters, cytokine receptors, transforming growth factor beta (TGF-β) and epidermal growth factor receptor (EGFR), which are involved in tumor formation and cell migration associated with cancer progression [54,55,56]. 

## 6. Sialylated N-Glycans

Sialylated glycans are the ligands of various proteins involved in crucial biological processes; cell surface sialylated glycans are engaged in the immune response, signal transduction and embryonic development. The sialic acid residues operate as receptors for specific ligands, such as siglecs and selectins [57,58]. Many reports have also stated that sialylated glycans are involved in oncogenesis and malignant progression [58]. The transformation of healthy cells into heterogeneous cancer cells is accompanied by the appearance of an abnormal sialylation pattern, which is reflected in a large group of sialylated glycoproteins secreted by tumor cells [58,59]. One of the decisive glycomic features associated with malignant and metastatic progression is the presence of N-glycans terminated with α2,6-N-acetylneuraminic acid (Neu5Ac) residues, controlled by the action of β-galactoside-α2,6-sialyltransferase I (ST6Gal-I). Expression of this enzyme is modified in various malignancies, including prostate, breast and ovarian cancer [60,61,62]. The presence of α2,6-linked sialic acids on tumor cells is crucial because α2,6-sialylation can execute alternative biological outcomes compared to α2,3-sialylation. One accurate example is the impact of α2,6-sialylation on galectin-dependent cell behaviours. Numerous studies indicate that α2,6-sialylation of galactose acts as a general inhibitor of galectin binding, in contrast to α2,3-sialylation with various binding effects to some particular galectins. Therefore, glycans capped with α2,6-sialic acid globally act as a significant negative regulator of many key galectin functions. One of the important activities of cell surface α2,6-sialyation is to suppress the binding of pro-apoptotic galectins, at the same time inducing cancer cell survival [63,64]. 

Additionally, elevated sialylation in cancer can be related to the formation of polysialic acid, which is associated with several types of cancers and is often present in high-grade tumors [58,65]. Other main sialylated antigens associated with cancer are sLe^a^ and sLe^x^. These have been shown to be remarkably expressed in many malignant tumors, including hepatic, renal and breast cancers [66]. Additionally, sLe^x^ antigen expression level has been associated with a diminished chance of survival for prostate cancer patients [67]. SLe^x^ and its isomer sLe^a^ are crucial recognition determinants for selectins, vascular cell adhesion molecules belonging to a large family of C-type lectins. In the process of inflammation, selectins mediate the first stage of leucocytes’ adhesion to the endothelium during leucocyte extravasation [68]. In cancer cells, sLe^x^ interactions with selectins regulate the metastatic process by tethering platelet-tumor cell emboli and promoting their arrest on the endothelium, therefore contributing to malignant behaviour and the progression of metastasis [69]. 

Over the years, PSA glycosylation changes have been analysed by mass spectrometry, and the level of α2,3-linked sialic acid was reported to be remarkably different in prostate cancer patients compared to a control group, stressing the importance of PSA sialylation in distinguishing cancer patients from healthy men (Figure 1B) [14,16,70]. In patients with prostate cancer, serum PSA contains increased levels of α2,3-linked sialic acid connected to the terminal galactose residue compared to healthy individuals [14,71]. Additionally, Ohyama et al. detected that binding of prostate cancer related PSA to *Maackia amurensis* lectin specific for α2,3-linked sialic acid was more intense than binding of PSA from healthy individuals [72]. Serum glycoproteins of prostate cancer patients showed increased levels of α2,3-linked sialic acid in relation to serum of patients with benign prostatic hyperplasia. The authors suggested that this feature can be used to predict the Gleason score with greater sensitivity and specificity than PSA concentration, used to date [73]. 

In metastatic prostate cancer two main forms of PSA were detected in the serum: a free form and alpha-1-antichymotrypsin (ACT)-complexed PSA. Both were characterized by mostly sialylated biantennary glycan structures, but the presence of several multi-antennary complex structures was also observed [14]. Yoneyama et al. developed a more sensitive diagnostic PSA test, in which they used a magnetic microbead-based immunoassay, that directly measured the amount of α2,3-linked sialic acid on the free serum PSA. The new assay showed a sensitivity of 95% and a specificity of 72% in a measurement carried out on a cohort of over 300 serum samples, from patients who underwent biopsy, including 138 PCa and 178 non-PCa patients with a PSA level less than 10.0 ng/mL [16]. This solution was more sensitive and accurate than the conventional PSA level and the free PSA-percent tests in the diagnosis of prostate cancer. Another study conducted in 13 BPH and 34 prostate cancer patients’ sera (including 17 Gleason grade 5 and 17 Gleason grade 7 samples), which considered N-glycans from the whole serum glycoproteins, indicated that tetra-antennary tetra-sialylated N-linked oligosaccharide levels were higher in the serum samples from patients with Gleason score 7 compared to Gleason score 5. Conversely, levels of tetra-antennary tetra-sialylated glycans with terminal fucose and tri-antennary tri-galactosylated glycans were significantly lower in serum from patients with a Gleason score of 7 compared to a Gleason score of 5 [73]. Moreover, the detection of α2,3-linked sialic acid PSA glycoforms combined with the PHI in a cohort of 79 patients showed 100% sensitivity and 94.7% specificity. The proposed analysis proved superior to PSA to distinguish aggressive prostate cancer from low-risk and benign disease (Table 1) [74].

The most recent study considering whole serum N-linked glycans in various prostate cancer stages aimed to investigate whether an altered glycosylation pattern could differentiate distinct forms of prostate cancer, including indolent, significant, and aggressive PCa. N-glycan profiling was performed on 117 prostate cancer serum samples using an automated, high-throughput analytical platform, which exploits ultra-performance liquid chromatography for high resolution separation of N-glycans. The results revealed a decreased amount of tri-antennary, tri-galactosylated tri-sialylated glycans with and without core fucose residues, corresponding to the transition of PCa from the indolent stage through significant and aggressive disease. Additionally, an increase in hybrid, oligomannose, bisecting GlcNAc and monoantennary glycans was observed (Table 1) [78].

Another interesting investigation regarded the main sialylated PSA glycoforms from the serum of aggressive PCa patients in relation to standard PSA from seminal plasma of healthy men. Exploiting *Sambucus nigra* affinity chromatography, the α2,6-linked sialic acid glycoforms were separated from α2,3-linked glycoforms, then PSA N-glycans were analysed by hydrophilic interaction liquid chromatography. The results indicated that levels of serum PSA sialylated glycoforms bearing GalNAc moieties (LacdiNAc) were raised in aggressive PCa patients. Concomitantly, levels of disialylated core fucosylated biantennary structures with α2,6-linked sialic acid, which were previously indicated as major PSA glycoforms characteristic for standard PSA from healthy men, were significantly lowered in aggressive PCa (Table 1) [79].

## 7. Fucosylated N-Glycans

Fucosylated glycans are synthesized by a series of fucosyltransferases. The modification generally occurs as core fucosylation and terminal fucosylation, the latter including specific Lewis blood group antigens such as Le^x^, Le^y^, Le^a^, and Le^b^. The core fucosylation of protein relies on addition of a fucose residue to the innermost N-acetylglucosamine (GlcNAc) residue of N-glycans via an α1,6-linkage, and is catalysed by fucosyltransferase Fuc-TVIII (Figure 1C), encoded by the *FUT8* gene [80]. Altered expression of *FUT8* and *FUT6* is an important feature in several cancers such as high-grade prostate cancer and breast cancer [80,81]. The *FUT6* gene encodes α1,3-fucosyltransferase and is upregulated in distant metastases. It was also reported that the product of this gene can participate in metastasis to bones [82]. Furthermore, expression of *FUT6* might trigger prostate cancer cell trafficking through an E-selectin-dependent mechanism [83,84]. Overexpression of *FUT8* has been recently linked with aggressive and castrate-resistant prostate cancer, as well as being associated with a poor prognosis for patients [85,86]. More recent data indicate that *FUT8* is involved in controlling the function of cancer cell membrane receptors [87]. Core fucosylation transforms cell surface molecules as well as the tumor microenvironment, and thus the extracellular matrix and growth factors, supporting cancer progression. *FUT8* promotes cancer cell invasiveness by remodelling the core fucosylation of the TGF-β receptor [88,89], as the presence of core fucose strongly affects the binding affinity of the TGF-β receptor and thus TGF-β induced epithelial-mesenchymal transformation (EMT) [90,91].

In the serum of prostate cancer patients increased core fucosylation of glycans has been found, compared to patients with BPH as well as men without known prostatic disease. These findings suggest that core fucosylation is associated with disease progression [73,92,93]. Saldova et al. established a high-throughput HPLC assay and used it for quantitative analysis of N-glycans from the whole serum glycoproteins of BPH and prostate cancer samples. Additionally, the samples were split into low and higher grade Gleason groups. A significant increase in core-fucosylated biantennary glycans in prostate cancer related to BPH was observed, but no changes in these glycans were associated with Gleason scores (Table 2) [73].

Studies utilizing the two prostate cancer cell lines LNCaP and PC3 have shown that fucosylation of glycans is increased in the rapidly proliferating and more invasive PC3 cell line compared to the slow growing, less invasive LNCaP cell line [85]. Wang et al. detected expression of α1,6-fucosyltransferase solely in PC3, and not in LNCaP cells. Afterwards the authors found that *FUT8* expression was elevated in metastatic PCa tissues compared to normal prostate tissues. Using PC3 and LNCaP cells as models, they also confirmed that *FUT8* overexpression in LNCaP cells increased PCa cell migration, while the silencing of *FUT8* expression in PC3 cells reduced cell motility. These results suggest the association of *FUT8* with aggressive prostate cancer (Table 2) [91,101].

In a recent study Clark et al. evaluated the effect of altered α1,6-fucosyltransferase expression on extracellular vesicles (EVs) in a prostate cancer cell model. They found that increased cellular expression of *FUT8* can reduce the number of vesicles secreted by prostate cancer cells and simultaneously enhance the protein abundance correlated with cell motility and prostate cancer metastasis. Overexpression of *FUT8* can also cause changes in glycans presented on EV-derived glycoproteins [101]. Wang and co-workers developed a quantitative lectin immunoassay using *Lens culinaris* agglutinin (LCA) and *Aleuria aurantia* lectin (AAL) to evaluate the level of PSA fucosylated glycoforms in serum samples from prostate cancer patients with different Gleason scores. The results demonstrated that both LCA and AAL immunoassays identified an increased level of fucosylated serum PSA. These results were concomitantly correlated with higher Gleason scores. Finally, the authors concluded that the determined fucosylated PSA forms could be valuable biomarkers to differentiate between aggressive and non-aggressive prostate cancer (Table 2) [94].

## 8. Branched N-Glycans

During malignant transformation and cancer progression, a frequently occurring change in the glycosylation pattern is the increased expression of complex β1,6-branched N-glycans [102]. The raised level of GlcNAc-branching N-glycans is a result of increased activity of mannoside N-acetylglucosaminyltransferase 5 (GnT-V), which is encoded by the *MGAT5* gene, often activated in cancer cells [59]. Activity of GnT-V leads to the formation of complexed tri- and tetra-antennary structures (Figure 1A), which can affect the stability, functional activity and half-life of proteins, as well as membrane dynamics [61,103]. Furthermore, branched N-glycans can be modified via β1,4-galactosyltransferases (β1,4-GalTs), thus elongated with poly-N-acetyllactosamine repeats, and finally capped with sialic acid or fucose. Poly-N-acetyllactosamine moieties are ligands for galectins, a family of evolutionarily conserved carbohydrate-binding proteins. These lectins bind glycans with high avidity and affinity, forming multivalent galectin-glycan lattices that control glycoprotein clustering and endocytosis, to regulate receptor signaling and activation [104]. Galectins play an essential role in cancer, participating in neoplastic transformation, survival of cancer cell, angiogenesis and tumor metastasis [59,105]. In contrast to the function of GnT-V, GnT-III catalyses the attachment of bisecting GlcNAc N-glycans via a β1,4-linkage (Figure 1C), inhibiting further conversion and extension of the glycan, as seen in β1,6-branched structures. GnT-III counteracts the role of GnT-V in the neoplastic process by participating in the inhibition of cancer metastasis [59].

Numerous studies have indicated that N-acetylglucosaminyltransferase V is an important tumorigenesis- and metastasis-associated enzyme in prostate cancer [106]. Lange et al. reported that β1,6-GlcNAc tri- and tetra-branched N-glycans were increased in cell line xenograft mouse models of prostate cancer (Table 2) [97]. Additionally, it was found that patients with castration-resistant prostate cancer had both overexpressed transcription levels of N-glycan branching enzymes and increased tri- and tetra-antennary N-glycans [91,96]. Slightly different observations were reported for direct expressed-prostatic secretion (EPS) and EPS urine samples. A decline in the amount of tri- and tetra-antennary glycans in advanced prostate cancer samples with a Gleason score of 8 and 9 was detected, and at the same time many biantennary structures with a bisecting-GlcNAc residue were observed, suggesting antimetastatic activity of GnT-III [95]. In some recent research it has been emphasized that changes in branched N-glycans can help to distinguish between BPH and prostate cancer, and furthermore increased levels of serum tri- and tetra-antennary N-glycans can be clinically useful for predicting castrate-resistant prostate cancer (Table 2) [96]. In a recent study, tetra-antennary N-glycans were identified as part of a biomarker panel to refine the distinction of patients with indolent and aggressive prostate cancer and predict patient survival [107].

## 9. LacdiNAc Structures

In general, LacdiNAc structures are rarely observed in normal mammalian cells, while their frequency is significantly increased in prostate, ovarian, and pancreatic cancers [98,108,109]. The terminal modification of N-glycans results from the β4-linkage of N-acetylgalactosamine to N-acetylglucosamine to form the LacdiNAc unit (GalNAcβ1→4GlcNAc). Two human β4-N-acetylgalactosaminyltransferases (β4GalNAcTs)—β4GalNAcT3 and β4GalNAcT4—are involved in biosynthesis of the LacdiNAc motif (Figure 1A). Although the enzymes have high sequence homology and similar substrate specificities, they present different tissue distribution: the *B4GALNT3* gene is mainly expressed in the human testis, stomach and colon, while the *B4GALNT4* gene is expressed in the human brain and ovary [109]. 

LacdiNAc structures have been documented for PSA N-glycans purified from seminal plasma of healthy individuals and from the human prostate cell line LNCaP. The amounts of the LacdiNAc moieties were increased in PSA obtained from prostate cancer cells [71,109]. Furthermore, the occurrence of LacdiNAc groups on PSA has been observed in numerous studies using *Wisteria floribunda* agglutinin (WFA) [98,99,100,110]. Interestingly, in prostate cancer the upregulation of β4GalNAcT4 was observed, while no changes were noted for β4GalNAcT3 [99,109]. Additionally, a correlation between enzyme upregulation and overexpression of LacdiNAc epitopes in prostate cancer derived PSA was found (Table 2) [99]. When prostate cancer and benign prostatic hyperplasia PSA were compared, BPH patients’ glycoproteins contained predominantly terminally sialylated, complex-type biantennary N-glycans, whereas PSA from patients with prostate cancer presented an increased amount of similar biantennary complex-type glycans, but with one LacdiNAc unit, only partially sialylated [109]. Haga and co-workers observed that abundance of multiple sialylated LacdiNAc structures was significantly upregulated in PCa patients compared to the BPH group, and further established a new, highly sensitive PCa-specific diagnostic model: the “PSA G-index”. It is based on the relative abundance of the two di- and tri-sialylated LacdiNAc glycoforms. Both above-mentioned PSA glycoforms showed a significant correlation with Gleason scores. In the same study, histochemical staining analysis with WFA lectin showed that PCa cells overexpressed glycoproteins containing LacdiNAc moieties (Table 2) [98]. The low sensitivity of tests developed so far encourages the use of a combination of several markers in the assay, suggesting that such a combination could ultimately constitute a biomarker panel for prostate cancer detection, as proposed by Yoneyama et al. To identify clinically significant prostate cancer (CSPC), they evaluated the amount of LacdiNAc-glycosylated prostate-specific antigen (LDN-PSA) and LDN-PSA normalized by prostate volume (LDN-PSAD). During the experiment, they measured LDN-PSA, total PSA, and ratio of free PSA to total PSA values in 718 men who underwent a prostate biopsy and in 174 prostate cancer patients who underwent radical prostatectomy. In the cohort of prostate biopsy patients LDN-PSAD demonstrated significantly higher clinical performance to discriminate CSPC compared with LDN-PSA, PSAD, total PSA and free/total PSA ratio. The authors concluded that LacdiNAc-glycosylated PSA is significantly more efficient than the conventional PSA test in identifying patients with CSPC [111].

## 10. Truncated O-Glycans

Biosynthesis of O-glycans is initiated by transfer of a single N-acetylgalactosamine residue to serine or threonine by polypeptide N-acetylgalactosaminyltransferases ppGalNAc-Ts (GALNTs), to form a simple Tn antigen [112]. In subsequent stages enzymatic extension of Tn antigen with galactose builds core 1 O-glycans (T antigen), which can be further extended with β1,6-N-acetylglucosamine to produce core 2 O-glycans (Figure 1E,F). GCNT1 is reported to be involved in the formation of core 2 branched O-glycans and in synthesis of the cancer-associated antigen sLe^x^ [113,114]. Increased GCNT1 expression has been linked to prostate cancer progression and is a predictor of recurrence after radical prostatectomy [114,115]. Expression of both GCNT1 and the sLe^x^ antigen is controlled by androgens in prostate cancer cells. Moreover, sLe^x^ is the major sialylated antigen related to poor prognosis and metastasis in PCa [48,113].

Alternatively, attachment of the N-acetylneuraminic acid molecule to the Tn antigen can generate sialo-Tn antigen (sTn) (Figure 1D). Formation of the sTn antigen is regulated by expression of sialyltransferase ST6GalNAc-I. Such modifications are frequently dysregulated, often occur during the neoplastic transformation process and are associated with numerous malignancies, including breast, colorectal, ovarian and gastric cancers [116,117]. Munkley et al. found that expression of the sialyltransferase ST6GalNAc-I and the cancer-associated sialyl-Tn antigen is regulated by androgens in prostate cancer cells, and is involved in reducing cell adhesion, leading to transformation towards a more mesenchymal-like cell phenotype in a mouse model of prostate cancer, during a process termed epithelial-mesenchymal transition (EMT) [118,119]. This fact is worth emphasizing, as the sTn antigen was detected in half of all high-grade prostate cancer tissue samples from patients with diagnosed prostate disease [118,120]. Additionally, ST6GalNAc-I expression was found to be increased in primary prostate tumors and decreased in metastatic tissue compared to benign prostate tissue. Several studies have shown that increased expression of sialylated Tn antigens inhibits formation of a solid tumor mass and promotes cell detachment from the tumor (Table 3) [118,121]. These results indicate that ST6GalNAc-I may play a significant role in tumor cell invasion and migration [118,119]. In earlier research of Arai and co-workers, the presence of sTn in the serum or tumor tissue biopsy was correlated with cancer progression and worse survival outcomes in prostate cancer patients. Additionally, the expression of sialyl-Tn MUC-1, a protein that plays a protective role for the mucosal epithelial surface [122], was negatively correlated with survival outcomes and positively correlated with higher serum PSA levels (Table 3) [123].

Some studies have also examined the role of core-2-O-linked sLe^x^ in prostate cancer, with a particular focus on metastatic disease [113,125,126]. Overexpression of sLe^x^ has been correlated with an inferior prognosis, among patients with metastatic prostate cancer, and with castration-resistant, aggressive prostate cancer [113]. Highlighting the role of O-glycans, Chen et al. found an increase in core-2-O-linked sLe^x^ antigens on PSA, MUC-1, and prostatic acid phosphatase (PAP) proteins in malignant relative to non-malignant prostate tissues, in patients who had undergone radical prostatectomy [113]. Many prostate cancer clinical trials have focused on mucin-1 (MUC-1), a single pass membrane protein, for which both the expression level of MUC-1 and its glycosylation were frequently altered [127]. An elevated MUC-1 level was detected in 58% of primary tumor tissues and 90% of lymph node metastases, but not in healthy prostate or benign prostate tissues [128].

A recent study by Bai et al. showed that sialyl-T antigen was extensively elevated in all prostate cancer cell lines (VCaP, LNCaP, DU145, PC-3) compared to normal RWPE-1 cells, and it was particularly visible in PC-3 cells. Further research focused on examination of ST3Gal-I function in PC-3 cells; ST3Gal-I silencing studies indicated that ST3Gal-I is correlated with migration, proliferation and apoptosis of PC-3 cells. Subsequently, in vivo studies showed that decreased ST3Gal-I expression was associated with a reduction in tumor size in a xenograft mouse model, demonstrating that sialyl-T antigen could be considered as a biomarker for the prognosis of prostate cancer metastasis (Table 3) [124].

## 11. Conclusions and Future Perspectives

Glycoproteins are often considered as prognostic biomarkers for cancer diagnosis and monitoring of tumor progression, as well as predictive biomarkers for disease recurrence [129]. Researchers are constantly working on new approaches to the early diagnosis of prostate cancer, risk prediction and disease treatment, and emphasizing that glycans can be a source of new, non-invasive biomarkers. Glycoprotein markers are characterized by high heterogeneity, which results from multiple glycosylation sites and glycosylation patterns, and it has been emphasized that these features may significantly alter the selectivity of these molecules. Therefore, an attempt to broaden the knowledge of cancer-specific glycan structures and glycosylation sites, and then compare their patterns within a healthy population, can provide the first key step towards determining the importance of glycosylation in the diagnostic process. In view of these aspects, considerable efforts are still being made to standardize a glycomics protocol and implement modern, high-throughput mass spectrometry technology [77,130,131,132].

The biomarker most frequently used in prostate cancer diagnosis is prostate-specific antigen, but its limitations due to relatively low specificity restrict its use in screening tests and reduce the diagnostic potential [59,133]. The diminished sensitivity of these tests for early prostate cancer screening, along with the emergence of novel methods and technologies for glycan analysis, prompted the search for novel biomarkers based on the detection and identification of specific glycoforms of individual glycoproteins. This approach may lead to the establishment of new biomarkers with higher specificity for early cancer detection or for diagnosis in precancerous lesions [134,135]. Such research has become possible to carry out using newly developed, high-throughput platform technologies, which additionally enable the effective analysis of large sample cohorts [79,134]. Numerous studies have investigated whether a cancer-specific glycosylation pattern on PSA can be used to differentiate between BPH and PCa [75,133]. The most recent research on glycan composition in order to identify PCa and establish a prognosis has been conducted on diverse biological material, including serum [78], urine [77,136], expressed prostatic secretion urine [137], formalin-fixed, paraffin-embedded (FFPE) tissues [138,139], cell lines [124,140], and exosomes [141].

Several studies have reported altered glycosylation, mainly both sialylation and fucosylation, in PSA as specific biomarkers of prostate cancer that are able to distinguish it from benign prostatic hyperplasia [73,133]. A crucial change in prostate cancer glycosylation is the increased level of sialylation, as well as the overexpression of cancer-related sialoglycans. Abnormal sialylation is evidently associated with tumor growth, invasion and enhanced cell survival and the onset of metastasis. Additionally, an increased level of the α2,3-sialylated isomer of PSA was noted as a distinctive feature of aggressive prostate cancer [16,70,76]; thus, analysis more targeted at aberrant sialylation is likely to be of significant therapeutic value [142]. Hence, a high-performance assay has recently been developed that allows for differentiation of α2,3- and α2,6-linked sialic acid isomers of PSA in urine [136], and subsequently a mass spectrometric method for distinguishing α2,3- and α2,6-sialoglycopeptide isomers in seminal plasma PSA was optimized [143], with good prospects for application of both methods in the diagnosis of prostate cancer. Thus, high-performance analysis of glycopeptides presenting prostate cancer-associated glycans has recently opened up new avenues for the discovery of glycoconjugates and glycoforms for the future emergence of cancer biomarkers with potential clinical applications [144].

In summary, composition of glycoproteins and glycans is likely to play an important role in both non-malignant prostate cancer and in prostate cancer. Hence in recent years, impressive progress in understanding their composition and function has been achieved. Numerous studies in this area have contributed to the emergence of glycans as promising biomarkers, highlighting their use in clinical settings as attractive targets for prostate cancer treatments [48,59,145].

## Figures and Tables

**Table 1:** Summary of sialylated N-glycan alterations in prostate cancer.

Type of Glycans	Structure	Sample Groups	Main Results	Author Year References
α2,3-sialylated	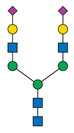	Serum of PCa patients vs. serum of non-PCa patients	Serum α2,3-linked sialic acid of PSA in PCa group was significantly higher than in non-PCa group	Yoneyama 2014 [16]
		PCa serum vs. control serum (healthy men)	Level of α2,3-linked sialic acid of PSA from prostate cancer serum was increased compared to healthy individuals	Pihikova 2016 [70]
		BPH serum vs. PCa serum	Significant increase in α2,3-linked sialic acid from total serum glycoproteins in PCa group comparing with BPH group was detected	Saldova 2011 [73]
		BPH serum, low-risk PCa serum, intermediate-risk PCa serum, and high-risk PCa serum samples	There was a significant increase of α2,3-sialylated PSA in the group of high-risk PCa patients compared with the intermediate-risk PCa, low-risk PCa, and BPH groups	Llop 2016 [75]
		BPH serum vs. PCa serum	Serum % α2,3-sialic acid of PSA was significantly higher in patients with PCa compared to BPH patients	Ishikawa 2017 [76]
		BPH serum vs. PCa serum	The combination of % α2,3-SA PSA and PHI differentiates high-risk PCa patients from the low and intermediate-risk PCa patients	Ferrer-Batalle 2017 [74]
		PCa urine samples with varied Gleason scores	Highly sialylated urinary N-glycans were upregulated in metastatic cancer patients	Yang 2017 [77]
Tetra-antennary tetrasialylated	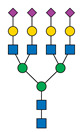	BPH serum vs. PCa serum (17 Gleason grade 5 and 17 Gleason grade 7 samples)	Tetra-antennary tetra-sialylated glycans were increased in the serum samples from patients with Gleason score 7 compared to Gleason score 5	Saldova 2011 [73]
Tri-antennary, tri-galactosylated tri-sialylated with and without core fucose residue	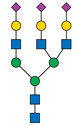	Indolent, significant, aggressive PCa according Epstein’s criteria	A decreased amount of tri-antennary, tri-galactosylated tri-sialylated glycans with and without core fucose residue corresponding to the transition of PCa from indolent state through significant and aggressive disease	Gilgunn 2020 [78]
Sialylated glycoforms bearing GalNAc moieties	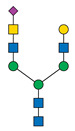	Serum PCa vs. standard SP PSA of healthy men	Serum PSA sialylated glycoforms bearing GalNAc moieties were heightened in aggressive PCa patients	Gratacós-Mulleras 2020 [79]

**Table 2:** Summary of fucosylated, bisected, tri- and tetra-antennary N-glycan alternations in prostate cancer.

Type of Glycans	Structure	Sample Groups	Main Results	Author, Year References
Fucosylated	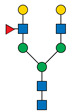 or/and 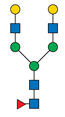	Healthy individuals serum vs. PCa patients serum	In the serum of PCa patients increased fucosylation of glycans compared to healthy individuals was found	Kyselova 2007 [91]
		BPH serum vs. PCa serum (17 Gleason grade 5 and 17 Gleason grade 7 samples)	A significant increase in core-fucosylation biantennary glycans in PCa serum relative to BPH was observed, no changes were associated with Gleason scores	Saldova 2011 [73]
		LNCaP-an androgen dependent cell line and PC3- an androden independent cell line	Fucosylation of glycans was increased in the rapidly proliferating and more invasive PC3 cell line relative to the slow growing, and less invasive LNCaP cell line	Shah 2015 [85]
		PC3 vs. LNCaP and metastatic PCa tissues vs. normal prostate tissues	*FUT8* expression was elevated in metastatic PCa tissues compared to normal prostate tissues *FUT8* overexpression in LNCaP cells increased PCa cell migration, while loss of *FUT8* in PC3 cells decreased cell motility	Wang 2014 [80]
		PCa serum samples with different Gleason scores	LCA- and AAL-immunoassays detected increased fucosylated PSA, results were correlated with higher Gleason scores	Wang 2019 [94]
Bisected	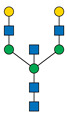	EPS-urine pools from aggressive PCa, indolent PCa and non-cancer urine samples	The presence of bi-antennary structures with bisecting-GlcNAc residue was increased with disease severity	Nyalwidhe 2013 [95]
Tri- and tetra-antennary	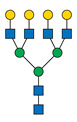	Serum samples from healthy men, BPH, early stage-PCa, PCa with ADT and CRPC patients	Tri- and tetra-antennary glycans were significantly higher in CRPC patients compared to the other groups	Ishibashi 2014 [96]
		Prostate cancer cells LNCaP, PC-3, LuCaP 23.1, and DU-145	*MGAT5B* gene overexpression was observed in all prostate cancer cell lines compared to normal prostate epithelium. β1,6-branched oligosaccharides were restricted to metastatic prostate cancer xenografts	Lange 2012 [97]
LacdiNAc motif	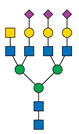	Normal seminal fluid and prostate cancer cells LNCaP	The amount of the LacdiNAc moieties was increased in LNCaP glycans in relation to structures obtained from normal SP PSA	Peracaula 2003 [71]
		Sera from PCa vs. BPH sera	Abundance of multisialylated LacdiNAc structures was significantly upregulated in the PCa patients compared to the BPH group	Haga 2019 [98]
		Seminal fluid, serum of BPH and PCa patients, and LNCaP cell line	In prostate cancer the upregulation of the β4GalNAcT4 was observed, and were correlated with the overexpression of the LacdiNAc groups for PCa-derived PSA	Fukushima 2010 [99]
		BPH serum vs. PCa serum	The LacdiNAc-PSA immunoassay allowed for distinction between PCa and BPH within the PSA gray zone The LacdiNAc-PSA determination revealed that levels of LacdiNAc-PSA were significantly higher in PCa sera than in BPH sera	Kaya 2015 [100]

**Table 3:** Summary of truncated O-glycan alternations in prostate cancer.

Type of Glycans	Structure	Sample Groups	Main Results	Author, Year References
sialyl-Tn (sTn) antigen	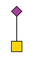	Prostate cancer specimens vs. normal prostate specimens	The presence of sialyl-Tn MUC-1 was correlated with cancer progression, positively correlated with higher serum PSA level in PCa patients, and negatively correlated with survival outcomes	Arai 2005 [123]
		primary and metastatic tumors	Expression of sTn was induced by androgens in prostate cancer cells and is mediated by ST6GalNAc-I. ST6GalNAc-I was significantly up-regulated in primary prostate carcinoma but relatively down-regulated in established metastatic tissue.	Munkley 2015 Munkley 2016 [118,119]
sialyl-T (sT) antigen	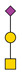	VCaP, LNCaP, DU145, PC-3 vs. RWPE-1 cells	sialyl-T antigen was extensively elevated in all prostate cancer cell lines (VCaP, LNCaP, DU145, PC-3) compared to normal RWPE-1 cells, especially in PC-3 cells	Bai 2020 [124]
core-2-O-linked sLex^x^ antigens	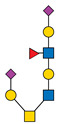	Malignant vs. non-malignant prostate tissues	An increase in core-2-O-linked sLe^x^ antigens on PSA, MUC-1, and PAP in malignant relative to non-malignant prostate tissues was observed	Chen 2014 [113]
